# Comparison of Four Methods to Assess Erosive Substance Loss of Dentin

**DOI:** 10.1371/journal.pone.0108064

**Published:** 2014-09-17

**Authors:** Falk Schwendicke, Geert Felstehausen, Clifton Carey, Christof Dörfer

**Affiliations:** 1 Department of Operative and Preventive Dentistry, Charité - Universitätsmedizin Berlin, Germany; 2 Clinic for Conservative Dentistry and Periodontology, Christian-Albrechts-University, Kiel, Germany; 3 Professor, Craniofacial Biology, School of Dental Medicine, University of Colorado, Denver, Colorado, United States of America; University of Toronto, Canada

## Abstract

Erosion of dentin results in a complex multi-layered lesion. Several methods have been used to measure erosive substance loss of dentin, but were found to have only limited agreement, in parts because they assess different structural parameters. The present study compared the agreement of four different methods (transversal microradiography [TMR], Confocal Laser Scanning Microscopy [CLSM], Laser Profilometry [LPM] and modified Knoop Hardness measurement [KHM]) to measure erosive substance loss in vitro. Ninety-six dentin specimens were prepared from bovine roots, embedded, ground, polished and covered with nail-varnish except for an experimental window. Erosion was performed for 1 h using citric acid concentrations of 0.00% (control), 0.07%, 0.25% and 1.00% (n = 24/group). Adjacent surfaces served as sound reference. Two examiners independently determined the substance loss. After 1 h erosion with 1% citric acid solution, substance losses (mean±SD) of 12.0±1.3 µm (TMR), 2.9±1.3 µm (LPM), 3.9±1.3 µm (KHM) and 17.0±2.6 µm (CLSM) were detected. ROC curve analysis found all methods to have high accuracy for discriminating different degrees of erosive substance loss (AUC 0.83–1.00). Stepwise discriminatory analysis found TMR and CLSM to have the highest discriminatory power. All methods showed significant relative and proportional bias (p<0.001). The smallest albeit significant disagreement was found between LPM and KHM. No significant inter-rater bias was detected except for KHM. LPM is prone to underestimate erosive loss, possibly due to detection of the organic surface layer. KHM was not found suitable to measure erosive loss in dentin. TMR and CLSM detected the loss of mineralised tissue, showed high reliability, and had the highest discriminatory power. Different methods might be suitable to measure different structural parameters.

## Introduction

Tooth erosion and erosive tooth wear have a continuously increasing prevalence, possibly due to an increased and more frequent consumption of acidic beverages [1], [2]. Studies to test erosive or preventive effects of various food or dental products are thus required and would best be performed clinically [3]. However, such research is limited by methodological and ethical considerations [4], with purposive exposure to harmful acidic attacks being unethical, monitoring of naturally occurring erosive substance loss requiring extensive follow-up periods, and clinical measurements often being restricted to the use of indices or the assessment of replica [5]. Thus, there is further need for in vitro or in situ studies, which will be designed not only according to the erosion to be modelled [5], but also the availability of measurement methods, which are required to be reliable, accurate and precise, but might also yield information not only regarding quantitative substance loss, but also the histology and further characteristics of the eroded lesion [6].

To date, most established methods have been thoroughly evaluated regarding the measurement of erosive effects in enamel [6]. There are substantially fewer studies investigating how erosive substance loss of dentin can be assessed, whilst the histopathology of enamel and dentin erosion is different [7]. Exposure to erosive agents removes the mineral content of dentin, whilst the organic components are retained. This eventually leads to a three-zoned tissue with a completely demineralised organic surface layer, a partially demineralised layer underneath and a sound dentin layer [8]. In addition, the demineralised organic layer is known to thicken during the erosive process until a steady thickness is reached, and an increasing thickness of the layer seems to decrease the loss of minerals in the layer beneath [9], [10]. Thus, it is thought that there is no bulk tissue loss as in enamel, but a complex and variable histology of eroded dentin [10].

Measurement methods to assess erosive substance loss in dentin should thus be evaluated and chosen according to both what they measure and how well (reliable, precise) they measure it. To assess erosive substance loss in dentin, neither transversal microradiography nor confocal laser scanning microscopy have been systematically compared with each other or established measurement methods like profilometry or modified hardness measurements. Therefore, the present study analysed the agreement between all four methods regarding measuring erosive substance loss of dentin, and evaluated their advantages, limitations and possible applications using standard erosion solutions.

## Materials and Methods

### Study design

We compared four different methods to measure erosive substance loss using a series of citric acid standards [11]. Substance loss was assessed in µm as described below. To assess inter-rater agreement of methods, two independent, calibrated examiners, who were blinded for the degree of substance loss, performed the analyses (GF, CD). Eventually, means of results obtained by both examiners were calculated and inter-method agreement assessed.

### Specimens preparation

For this study, we have used bovine incisors, which were obtained from a local slaughterhouse (VION, Bad Bramstedt, Germany, vionfoodgroup.com). The samples had originally not been collected for the purpose of research, but were obtained after slaughtering as described in previous publications, without ethical approval being seeked [12]. Extraction of incisors was performed under supervision of the local veterinary, and teeth were only utilized after being declared free of infectious diseases. From each of 32 bovine incisors of the second dentition, 4 axial root slices (5 mm height) were cut (Band Saw Exakt 300cl, Exakt Apparatebau, Norderstedt, Germany) and embedded in acrylic resin (Technovit 4071, Heraeus Kulzer, Hanau, Germany). Embedded slices were then ground flat into cubicles (Phoenix Alpha; Buehler, Düsseldorf, Germany), plan-parallelised, and polished (abrasive paper 1200, 2400, 4000 Exakt Apparatebau) under water cooling. Two thirds of the resulting exposed dentin surface (3×5 mm) were covered with a tape, and the complete dentin surface was covered with acid-resistant nail varnish (Maybelline, New York, USA). The tape was then carefully removed with a scalpel, resulting in a defined experimental area, which was then controlled using a stereomicroscope (Stemi, Zeiss, Jena, Germany).

### Erosive demineralisation

Citric acid solutions with concentrations of 0.07% (pH = 3.77±0.05, group 1), 0.25% (pH = 3.68±0.05%, group 2) and 1.00% (pH = 3.60±0.05, group 3) were prepared using citric acid (Carl Roth, Karlsruhe, Germany) and sodium citrate (Sigma, St. Louis, USA) according to international standards [11]. These standards are designed for validating methods to assess hard tissue erosion. One specimen from each tooth was then allocated to one of the solutions, or deionised water (pH 7.57, group 0), with 24 specimens being stored in 1 l of each agitated solution (50 rpm, Innova 40 orbital shaker, New Brunswick, Enfield, USA) for 60 min at 21.2°C. The pH values of all solutions were measured with a pH-meter (GMH, Greisinger, Regenstauf, Germany). After the treatment, the nail varnish was carefully lifted off the samples with a scalpel without contact to the dentin surface. The resulting eroded or sound dentin (groups 1–3 or 0, respectively,) of unprotected (experimental area) and protected surfaces (reference area) was then controlled once more (Stemi). Between analyses, specimens were stored in 0.08% thymolised water at 8°C.

### Modified Knoop hardness measurement (KHM)

For KHM, three impressions were made in each dentin specimen on the unprotected surface before the erosion process using a Knoop indenter (Zwick, Ulm, Germany) with a loading force of 4 kg. Images of indents before and after the erosion were obtained using a polychromatic light source (HAL 100, Zeiss) and a microscope (Axioplan 2, Zeiss) interfaced with a CCD camera (CFM 1312, Scion Corporation, Frederick, USA). Lengths of indents before (L_1_) and after the erosion process (L_2_) were measured (Axiovision Rel 4.6, Zeiss), and the substance loss height (ΔH) calculated, with ΔH being the differences of length (L_1_-L_1_ multiplied with 2tanα (α = 86.26°). Three indents were analysed per sample and examiner. To analyse the standard error of the microscopic analysis, we assessed a standard grit (Sci G400 C30). Repeated analysis of this grit for ten times resulted in a relative deviation (mean±SD) of 0.4±0.1% from the true value, and repeated analysis of the identical grit distance in a relative SD of 3.5%. Some samples could not be analysed after erosion, since indents were not clearly detectable anymore; the number of analysed samples is given within the results.

### Laser profilometry (LPM)

For profilometric analysis, a laser profilometer (μscan, NanoFokus, Oberhausen, Germany) with a confocal sensor (CF 4, NanoFokus) and a vertical solution of 0.5 µm was used. Profilometric tracings of experimental and reference areas were assessed with a scanning path perpendicular to the border between both areas. Scanning was performed with a speed of 1 mm/sec at 1000 Hz and a focus-object-distance of 4 mm. Resulting 2 mm tracings were analysed digitally (μsoft, Metrology, Saarlouis, Germany). For LPM and the following analyses, regression lines of reference and experimental areas were digitally plotted, and a line drawn perpendicularly to the reference regression line at the lesion border. The vertical distances of the crossings of this line with the regression lines was measured and assumed to equal the erosive substance loss. Each specimen was analysed five times by each examiner. Repeated analysis of a standard grit (Sci G400 C30) for ten times resulted in a relative deviation of −1.2±6.3% from the true value, and repeated analysis of the identical tracing in a relative SD of 1.3%.

### Confocal laser scanning microscopy (CLSM)

For CLSM, perpendicular transversal sections (500 µm) of specimens were prepared and plan-parallelised. A confocal laser scanning microscope (Axiovert 200, Zeiss) equipped with an argon/krypton laser (wavelength spectrum λ = 488–568 nm) was used in reflection mode at 63× magnification, a numerical aperture of 1.4 and an emission wavelength of 488 nm to analyse specimens. Measurements were performed digitally (LSM Image Browser, Zeiss), and each sample was analysed for five times by each examiner. Repeated analysis of a standard grit (Sci G400 C30) for ten times resulted in a relative deviation of 0.1±0.3% from the true value, and repeated analysis of the identical grit distance in a relative SD of 0.6%. Some samples were lost during the preparation process and could not be analysed.

### Transversal microradiography (TMR)

Thin plan-parallel transversal sections (100±10 µm) of specimens were prepared (Band Saw 300cl, Mikroschleifsystem 400 CS) and polished (abrasive Paper 1200, 2400 and 4000). A nickel-filtered copper X-ray source (PW 3830, Philips, Kassel, Germany) operating at 20 kV and 20 mA with a vertical tube (PW 3820, Pananalytical, Kassel, Germany) was used to obtain radiographs. Films (35 mm B/W positive, Fujifilm, Tokyo, Japan) were exposed for 10 s and developed under standardised conditions according to the manufacturer's recommendations. The microradiographs were analysed with a digital-imaging-system (CFM 1312, Scion) interfaced with a universal microscope (Axioplan 60318, Zeiss) at 100× magnification, and a personal computer (Axiovision Rel 4.6, Zeiss). Each microradiograph was analysed ten times by each examiner. Repeated analysis of a standard grit (Sci G400 C30) for ten times resulted in a relative deviation (mean±SD) of 0.2±0.2% from the true value, and repeated analysis of the identical tracing in a relative SD of 0.03%. Some samples were lost during the preparation process and could not be analysed.

### Statistical analyses

Statistical analysis was performed with SPSS 20 (IBM, Armonk, NY, USA). Normal distribution was controlled using Shapiro-Wilk-test. Values measured by each examiner were evaluated for inter-rater agreement, and mean values of both examiners used for further analysis. To assess the influence of the degree of erosive demineralisation and the measurement methods, analysis of variance was performed, and groups were compared using t-test, with Bonferroni adjustment to correct for multiple testing. Agreement between methods was assessed using Bland-Altman-plots, with the x- and y-axes representing the mean results obtained with both methods and the differences (Δ) between the methods, respectively. When analysing measurement data based on this approach, proportional bias is assumed to be present if the slope (R) of a calculated ordinary least square regression of the differences of the means is significantly different from 0. Fixed bias is indicated if the Δ is significantly different from 0 as indicated by the one-sample t-test or by 0 not being included within the 95% CI [13]. Receiver operating characteristics (ROC) curve and stepwise discriminant analysis were performed to evaluate the accuracy and power of measurement methods to discriminate between different groups of erosive substance loss, respectively. Level of significance was set at p<0.05.

## Results

Both measurement method and acid concentration had a significant influence on obtained results (p<0.001, ANOVA). There was no significant difference of detected substance loss in groups 1–3 between LPM and KHM (p>0.05, t-test/Bonferroni), whilst obtained results differed significantly between all other methods (p<0.05). TMR and CLSM yielded significantly higher results than LPM and KHM for groups 1–3 (Table 1). There were no significant inter-rater differences for all methods except KHM (Table 2). The latter and LPM showed proportional inter-rater bias as well (p<0.05). Detailed results for each examiner can be found within the appendix (Table S1).

**Table 1:** Erosive substance loss (in µm) according to different groups as measured using the four different methods.

Erosive substance loss in µm (mean±SD), N (in parentheses)				
Group	TMR	LPM	KHM	CLSM
0	0.11±0.04 ^Aa^ (24)	0.20±0.12 ^Ab^ (24)	0.09±0.13 ^Aab^ (24)	0.09±0.05 ^Aa^ (21)
1	3.64±0.30 ^Ba^ (23)	0.77±0.45 ^Bb^ (24)	0.93±0.49 ^Bbc^ (24)	4.84±0.98 ^Bd^ (21)
2	6.01±0.58 ^Ca^ (23)	1.79±0.51 ^Cb^ (24)	2.34±0.83 ^Cbc^ (22)	8.96±1.36 ^Cd^ (20)
3	11.97±1.25 ^Da^ (24)	2.87±1.25 ^Db^ (24)	3.85±1.33 ^Dbc^ (23)	17.03±2.55 ^Dd^ (20)

Obtained values differed significantly between groups regardless of the measurement method Different superscript letters indicate significant differences between measurement method, with uppercase letters indicating significant differences between groups, i.e. in columns (p<0.05, t-test/Bonferroni), and lowercase letters between methods, i.e. in rows.

Samples were immersed in solutions with 0% (group 0), 0.07% (group 1), 0.25% (group 2) or 1.0% (group 4) citric acid. Number of analysed samples per group is given in parentheses.

**Table 2:** Inter-rater agreement.

Method	N	Mean difference in µm (p-value^1^)	95% Confidence Intervals of mean difference		R (p-value)^2^
			Lower	Upper	
TMR	94	0.112 (0.084)	−0.016	0.245	0.117 (0.260)
LPM	93	−0.034 (0.180)	−0.084	0.016	0.214 (0.037)
KHM	91	0.368 (0.001)	0.218	0.518	0.234 (0.025)
CLSM	82	0.013 (0.851)	−0.122	0.148	0.099 (0.378)

1 based one-sample t-test in comparison with 0.

2 based on ordinary least square regression analysis.

Number of samples analysed per group (N), mean differences in µm, level of significance (in parentheses), 95% Confidence Intervals of this difference and slope (R) and level significance (p) of the linear regression line are given. Significant differences indicate relative bias between raters, whilst significant R-values indicate proportional bias.

**Figure 1:**
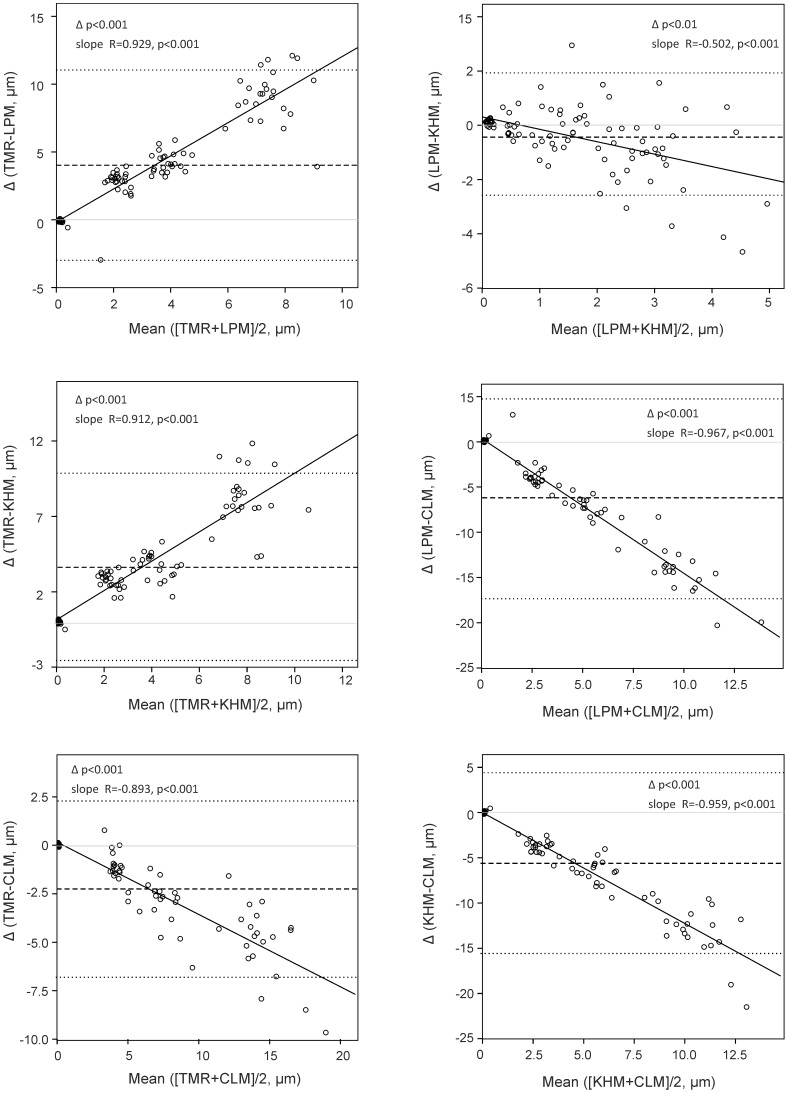
Bland-Altman plots. Differences (Δ) of measured substance loss between various methods were plotted against the mean measured values. Hatched line and dotted lines = mean and 95% Confidence Intervals of the difference. Solid grey line = zero-line. Solid black line = ordinary least square regression line. Relative bias between methods is indicated if pΔ<0.05. The slope (R) of the linear regression line is given, and proportional bias was assumed if p<0.05.

If compared with TMR, all other methods yielded results with significant relative and proportional bias (p<0.001). Whilst LPM and KHM resulted in significantly smaller and positively proportional values of substance loss than TMR, CLSM measures showed significantly higher values with negative proportional effects (p<0.001). Differences were smaller between TMR and CLSM than between TMR and LPM or KHM (Fig. 1). If compared with each other, LPM and KHM showed small but significant differences (p<0.001) and the lowest, albeit significant, proportional bias of all comparisons (Fig. 1). Differences between CLSM and KHM or LPM were significant with negative proportional bias (p<0.001, Fig. 1).

ROC curve analysis found TMR to have perfect accuracy for discriminating between different degrees of erosive substance loss, CLSM to have excellent to perfect accuracy, and KHM or profilometry to have good to excellent accuracy (Table 3). Stepwise discriminant analysis found TMR plus CLSM or TMR alone to have the highest discriminatory power for distinguishing between groups 1 and 2, or groups 2 and 3, respectively (Table S2).

**Table 3:** ROC curve analysis.

	Mean AUC (5/95% CI)	
Method	Group 1 vs. group 2	Group 2 vs. group 3
TMR	1.00 (1.00/1.00)	1.00 (1.00/1.00)
LPM	0.93 (0.86/0.99)	0.83 (0.71/0.95)
KHM	0.94 (0.88/1.00)	0.85 (0.73/0.96)
CLSM	0.99 (0.98/1.00)	1.00 (1.00/1.00)

The accuracy of different methods for discriminating between different degrees of substance loss (i.e. different groups) was evaluated via calculation of the area under the curve (AUC).

## Discussion

Assessing the histology of eroded dentin lesions using conventional measurement methods is challenging, since the remaining organic surface layer interferes with the detection of erosive substance loss, and is dimensionally instable depending on the environmental moisture [10], [14], [15]. This surface layer might explain the substantial disagreement we found between the different assessed measurement methods.

We measured significantly smaller substance loss values with LPM compared to CLSM and TMR. Non-tactile profilometry seems especially prone to detect the surface layer but not the border of demineralisation, as it is an optical measurement unable to distinguish between different mineralised qualities. In contrast, contact-profilometry was shown to measure deeper, since the stylus might enter the surface layer and scratch through it [16]. It remains unclear if this method allows reliable and complete penetration of the organic layer. Thus, LPM might be suitable to measure erosive loss if the whole remaining dentin including the organic layer was to be assessed. The latter was shown to be relatively resistant to abrasive forces [15], and its possibly erosion-protective function [9], [10] might be of interest for future studies. However, LPM had the lowest accuracy of all four methods to discriminate between different degrees of erosive loss. This is likely due to an increased thickness of the organic layer in case of more aggressive erosive attacks, thereby distorting the measurement results.

In comparison with CLSM or TMR, KHM measured lower substance loss and showed significant relative and proportional bias. Moreover, inter-rater agreement was limited, precision low, and discriminatory power inferior to TMR or CLSM. There are several possible reasons for these findings. In contrast to enamel, dentin is prone for drying and shows relaxation effects of up to 30% after the indentation [17]. Whilst we controlled the moisture of samples during measurement, relaxation effects remain likely, thereby decreasing the measured substance loss and introducing relative and, most likely, proportional bias. Moreover, measuring the relative indent length changes for evaluating erosive substance loss was shown to be of limited value, since the indentation deepens as well during erosive demineralisation [18]. We also detected a considerable loss of clear margins after the erosive attack, which increased the difficulty of correctly assessing the lengths of the indents. Similar difficulties due to mineral depositions around the margins have been reported [18]. In conclusion, despite being easy and cheap to perform, KHM has considerable limitations to assess erosive losses of dentin.

TMR was shown to measure with high inter-rater agreement even for relatively small substance losses, and had perfect accuracy for discriminating between different degrees of erosive loss. We found that even losses <10 µm can be reliably detected, confirming results from a previous study [19]. Thus, TMR seems to be superior for detecting smaller erosive losses, especially in dentin, to Longitudinal Microradiography [10], [20]. The reason for this difference between both microradiographic methods is the different specimens morphology: LMR-samples for measuring dentinal erosive loss require a thickness of 800 µm [10], and detect only erosive losses >25 µm due to high variability of results. TMR, in contrast, uses thin specimens with thicknesses of 50–200 µm, allowing to reduce detection limits to 10 µm or less. TMR further allows the detection of the mineralised front of the eroded dentin. However, it requires parallelised preparation and orthogonal alignment of samples as well as a close contact between sample and film to avoid surface blur [18]. Besides measuring the depth of substance loss, TMR is suitable for mineral loss analyses, allowing indirect assumptions of cross-sectional hardness as well. Since sample preparation is tedious and destructive, alternative techniques like Transversal Wavelength Independent Microradiography might be more suitable, especially for longitudinally monitoring erosive losses [21]. In conclusion, TMR was found to be a suitable method to assess dentin erosion even for smaller erosive losses, and had the highest discriminatory power of all assessed methods.

The use of CLSM to quantitatively assess erosive dentin loss has so far not been investigated. We showed high inter-rater agreement and precision of this method, and found CLSM to predict different degrees of substance loss with high accuracy. However, CLSM detected higher substance losses than all other techniques. This might be due to the lower detection limit of CLSM, and the three-dimensional tomographic analysis reduces possible decreasing effects of misalignment. Furthermore, CLSM allows sub-surface analyses and a comprehensive assessment of the erosive dentin lesions, and might be suitable for sequential quantitative measuring of natural surfaces as well. In conclusion, CLSM seems promising to non- or semi-destructively assess erosive losses. Possible reasons for the identified bias compared with TMR require further evaluation.

The present study used standardised erosive demineralisation solutions [11]. The 1.0% citric acid solution has been proposed as standard before [5], but acid concentrations below 1%, as used within the present study, result in relatively small substance losses only. These, however, were sufficient to detect relative and proportional bias between methods, and the discrimination between such small losses is of interest especially in in situ or clinical studies. It should be highlighted that from the four methods evaluated, only LPM can be used in clinical studies, requiring the use of replica: The other three methods assess either the mineral content or require standardized indents to be placed onto the dentin. Whilst TMR and CLSM might therefore have a place to evaluated erosive substance losses in *ex vivo* studies, KHM does not seem suitable for clinical application. Moreover, the use of flat, polished, bovine dentin in this study may have affected our results, with bovine dentin potentially being be more susceptible to erosion than human dentin due to a higher number of tubules per surface [22]. For all procedures, samples were stored moist before the measurement, and re-wetting performed regularly. It should be highlighted that for LPM and KHM, samples required blotting after the re-wetting to avoid reflection during profilometric and microscopic analyses. This may have introduced additional dimensional distortion.

In conclusion, all methods detected different degrees of substance losses. KHM might not be suitable to measure erosive loss in dentin. LPM seems to underestimate the complete substance loss, possibly due to detection of the organic surface layer. TMR and CLSM detected the loss of mineralised tissue and showed high reliability. Combining different measurement methods might be suitable to comprehensively assess eroded dentin lesions.

## Supporting Information

Table S1 **Erosive substance loss in each group as measured using the four different methods by examiner 1 and 2.** Samples were immersed in solutions with 0% (group 0), 0.07% (group 1), 0.25% (group 2) or 1.0% (group 4) citric acid.
(DOCX)

Table S2 **Stepwise discriminatory analysis.** Measurement methods were analysed for their discriminatory power to distinguish between groups 1 and 3, or groups 3 and 4. Model fit was analysed using canonical correlation coefficients (r) and Χ^2^-test. Standardized canonical discriminant function coefficients were reported for methods retained.
(DOCX)
