# Supplementary material for: Comparison of Four Methods to Assess Erosive Substance Loss of Dentin
Source: PLoS One. 2014 Sep 17;9(9):e108064. doi: 10.1371/journal.pone.0108064 (PMC4168231; doi:10.1371/journal.pone.0108064)
Supplement: Table S1 — Erosive substance loss in each group as measured using the four different methods by examiner 1 and 2. Samples were immersed in solutions with 0% (group 0), 0.07% (group 1), 0.25% (group 2) or 1.0% (group 4) citric acid. (DOCX) [file pone.0108064.s001.docx]

**Table S1: Erosive substance loss in each group as measured using the four different methods by examiner 1 and 2.** Samples were immersed in solutions with 0% (group 0), 0.07% (group 1), 0.25% (group 2) or 1.0% (group 4) citric acid.

Examiner 1

| Group | Erosive substance loss in µm (mean±SD), N (in parentheses) | | | |
| --- | --- | --- | --- | --- |
|  | TMR | LPM | KHM | CLSM |
| 0 | 0.10±0.06 | 0.27±0.12 | 0.11±0.16 | 0.08±0.06 |
| 1 | 3.63±0.56 | 0.74±0.51 | 1.12±0.53 | 4.91±0.92 |
| 2 | 6.20±0.65 | 1.79±0.54 | 2.47±0.76 | 9.01±1.44 |
| 3 | 12.04±1.31 | 2.76±1.30 | 4.23±1.44 | 16.94±2.66 |
|  | | | | |

Examiner 2

| Group | Erosive substance loss in µm (mean±SD), N (in parentheses) | | | |
| --- | --- | --- | --- | --- |
|  | TMR | LPM | KHM | CLSM |
| 0 | 0.11±0.08 | 0.13±0.20 | 0.07±0.13 | 0.09±0.06 |
| 1 | 3.66±0.28 | 0.81±0.40 | 0.74±0.58 | 4.77±1.10 |
| 2 | 5.83±0.65 | 1.79±0.50 | 2.21±1.10 | 8.90±1.35 |
| 3 | 11.90±1.34 | 2.76±1.30 | 3.46±1.36 | 17.11±2.51 |
|  | | | | |
