# Supplementary material for: Comparison of Four Methods to Assess Erosive Substance Loss of Dentin
Source: PLoS One. 2014 Sep 17;9(9):e108064. doi: 10.1371/journal.pone.0108064 (PMC4168231; doi:10.1371/journal.pone.0108064)
Supplement: Table S2 — Stepwise discriminatory analysis. Measurement methods were analysed for their discriminatory power to distinguish between groups 1 and 3, or groups 3 and 4. Model fit was analysed using canonical correlation coefficients (r) and Χ2-test. Standardized canonical discriminant function coefficients were reported for methods retained. (DOCX) [file pone.0108064.s002.docx]

**Table S2: Stepwise discriminatory analysis**. Measurement methods were analysed for their discriminatory power to distinguish between groups 1 and 2, or groups 2 and 3. Model fit was analysed using canonical correlation coefficients (r) and Χ²-test. Standardized canonical discriminant function coefficients were reported for methods retained.

|  | Group 1 vs. group 2 | Group 2 vs. group 3 |
| --- | --- | --- |
| Method | r=0.94; Χ²=73.9, df=2, p<0.001 | r=0.94; Χ²=73.1, df=2, p<0.001 |
| TMR | 0.78 | 1.00 |
| LPM |  |  |
| KHM |  |  |
| CLSM | 0.40 |  |
